# Matrilineal social rank is associated with patterns of attention to social scenes in male infant rhesus macaques

**DOI:** 10.1038/s41598-026-61421-w

**Published:** 2026-10-21

**Authors:** Aiden Ford, Natalie R. Pilgeram, Arick Wang, Maria C. Alvarado, Trina Jonesteller, Jabari Wesson, Kelly Bailey, Shaina Ginsberg, Prasanna Karur, M. Mar Sanchez, Jocelyne Bachevalier

**Affiliations:** 1 Department of Pediatrics, Emory University, School of Medicine, Atlanta, GA USA; 2 Marcus Autism Center, Children’s Healthcare of Atlanta, Atlanta, GA USA; 3 Emory National Primate Research Center, Emory University, Atlanta, GA USA; 4 Department of Psychology, Emory University, Atlanta, GA USA; 5 Department of Neuroscience and Behavioral Biology, Emory University, Atlanta, GA USA; 6 Department of Psychiatry & Behavioral Sciences, Emory University, School of Medicine, Atlanta, GA USA

**Keywords:** Nonhuman primate, Infant development, Social attention, Oculomotor movements, Matrilineal social rank, Longitudinal modeling, Evolution, Neuroscience, Psychology, Psychology, Zoology

## Abstract

Visual attention to others in our immediate environments serves as an adaptive mechanism for early learning across primate species. This study leverages longitudinal eye-tracking data from male infant rhesus macaques collected at up to 14 timepoints from birth to six months to chart visual attention patterns to social videos of unknown conspecifics or mother-infant dyads in a group setting. The distribution of oculomotor movements that index the extent of infant attention to the video stimuli – fixations, saccades, and inattention, i.e., data lost to offscreen looks or movement – stayed constant during this developmental period. Attention to different body areas – mouth, head and body - showed subtle and progressive changes from birth to six months, except for attention to the eyes of conspecifics, which showed two distinct periods of significant increase. The matrilineal social rank of the infant was also investigated as a social factor related to patterns of attention. Rank was significantly associated with the percent of time infants spent making saccades, such that low-ranking infants spent more viewing time making saccades than their high-ranking peers. This association was established by five weeks of age. Collectively, these results provide insight into processes of social attention related to social status in rhesus macaques during infancy.

**Supplementary Information:**

The online version contains supplementary material available at https://doi.org/10.1038/s41598-026-61421-w.

## Introduction

Visual attention to social information is a key mechanism by which we navigate the complex sensory environments of everyday life. Our preference for social information, particularly the eyes of other people over other features of the face and body, emerges in early infancy and is an adaptive behavior for social learning associated with the acquisition of social communication and perception skills^1–3^. Attention to caregivers’ facial expressions, eye contact, and vocalizations facilitate the developing infant’s attentional preferences for visual cues from the eye region and face of conspecifics (reviewed in^4^.

Cross-species studies of humans, great apes, and other non-human primates (NHPs) demonstrate that patterns of social attention, particularly to the eyes of others in the respective species, are evolutionarily conserved, both in terms of perceptual strategies and developmental changes^5,6^. This convergence indicates that patterns of social visual attention may reflect an adaptive mechanism for early learning across primate communities defined by complex social environments and interactions.

Rhesus macaques are an indispensable model system for human social development due to their altricial birth, dynamic social learning environment, and accelerated developmental timeline of approximately 1:4, e.g. one week in macaque development equating to four weeks in human development (reviewed in^7^). In addition, they allow for densely sampled behavioral and neuroimaging data challenging to obtain in human infants and minimization of confounding factors that are difficult to control in human studies (e.g., diet, education, and exposure to prenatal stress). Reported cross-species convergences between rhesus macaques and humans in brain and behavior development during infancy^7–11^, have established a strong foundation for continued investigation into the maturation and refinement of visual attention patterns and their neural correlates in rhesus macaque infants.

The first two postnatal months (eight weeks) are a period of key transition in social attention preferences in infant rhesus macaques. By four weeks of age, infant macaques reliably prefer face-like schematic configurations over other patterns^12^, and around 6–8 weeks, they exhibit a preference switch from initially attending to direct gaze^13^ and conspecific faces (other macaques) to attending more to averted gaze and heterospecific (other primate) faces^14^. From 5-6-weeks, infant macaques also begin to decrease the amount of time they spend attending to the eyes of other macaques after exhibiting a consistent increase from birth^6,15^- a developmental pattern strikingly convergent with human infants^16^. These transitions in social visual attention may be linked with changes in the infants’ social environment, specifically a growing independence that draws infants away from predominantly close contact, face-to-face interactions with their mother into interactions with same-age peers and other members of their larger social group.

Rhesus macaques live in large, multigenerational, polygynous, and matriarchal societies^17–19^, organized in matrilines with highly hierarchical and stable social status. Among macaques, rhesus macaques are considered a despotic, low tolerance species^20^, in that they exhibit stricter adherence to family-linked social dominance hierarchies. Thus, the social environment and experiences of an infant macaque are influenced from the moment of birth by its mother’s social status/rank within the larger society^21–23^, which is conferred to the infant. Comparative studies between rhesus macaques and Barbary macaques *(Macaca sylvanus*; classified as a more tolerant species) show that both species exhibit equal performance on social cognitive tasks^24^, but that more individual heterogeneity is present in rhesus macaques^24^ and that rhesus macaques exhibit less gaze following, i.e., a key social cognitive skill, over the course of their lifespan. Levels of gaze following in juvenile development between the two species are comparable^25^.

Individual social status affects how infant rhesus macaques visually engage with social scenes and the faces of others. At two months of age, high-ranking male infants look more at the expressive faces of conspecifics than low-ranking infants^26^, and by nine months high-ranking infants spend more time attending to threatening faces than low-ranking infants^27^. The authors concluded that this pattern represented a greater vigilance for threat, but it may also indicate a greater comfort engaging with threatening faces given that high-ranking infants are in relatively protected positions within social groups. More recently, our group found in an exploratory analysis that lower social rank was associated with an earlier increase in attention to the eyes of conspecifics with neutral expressions, suggesting that infant macaques across the range of social ranks differentially process critical social cues in their environment^15^.

Prior research has established that patterns of social visual attention change as infant rhesus macaques mature, with inflection points emerging relative to key transitions in infant engagement with their broader social groups. However, the majority of studies have characterized these changes under constrained experimental paradigms, specifically the use of static photographs or explicitly valanced stimuli. Densely-sampled, longitudinal studies of attention patterns to naturalistic social scenes are still needed to map fine-grained temporal change in attention at the individual level and to explicitly test how environmental factors, like social rank, relate to individual variability in trajectories of social learning.

Motivated by the age-varying effects of social rank on the development of social-visual attention processes demonstrated by our 2023 study^15^, this study further investigates the development of basic attentional processes in infants rhesus macaques from birth through six postnatal months with the following aims: (1) map developmental change in visual attention to social content – the eyes, mouth, head, and body of conspecifics – and oculomotor movements – saccades and inattention, i.e., data lost to movement or offscreen looks – in a large sample of infants (*N* = 39) with a diverse range of social ranks from the most dominant (“alpha”) to the most subordinate (“omega”) families and (2) test the predictive relationship between developmental trajectories of social-visual attention and oculomotor behavior, which reflect how infants intentionally allocate their visual resources to navigate and learn from their surrounding social environments, and the matrilineal social rank of the infant, i.e., their rank as determined by the matriline in which their mother is embedded. We hypothesize that visual attention patterns will be associated with infant social rank such that low-ranking infants look more to the eyes of others and utilize different visual strategies than their high-ranking peers.

## Results

**Fig. 1:**
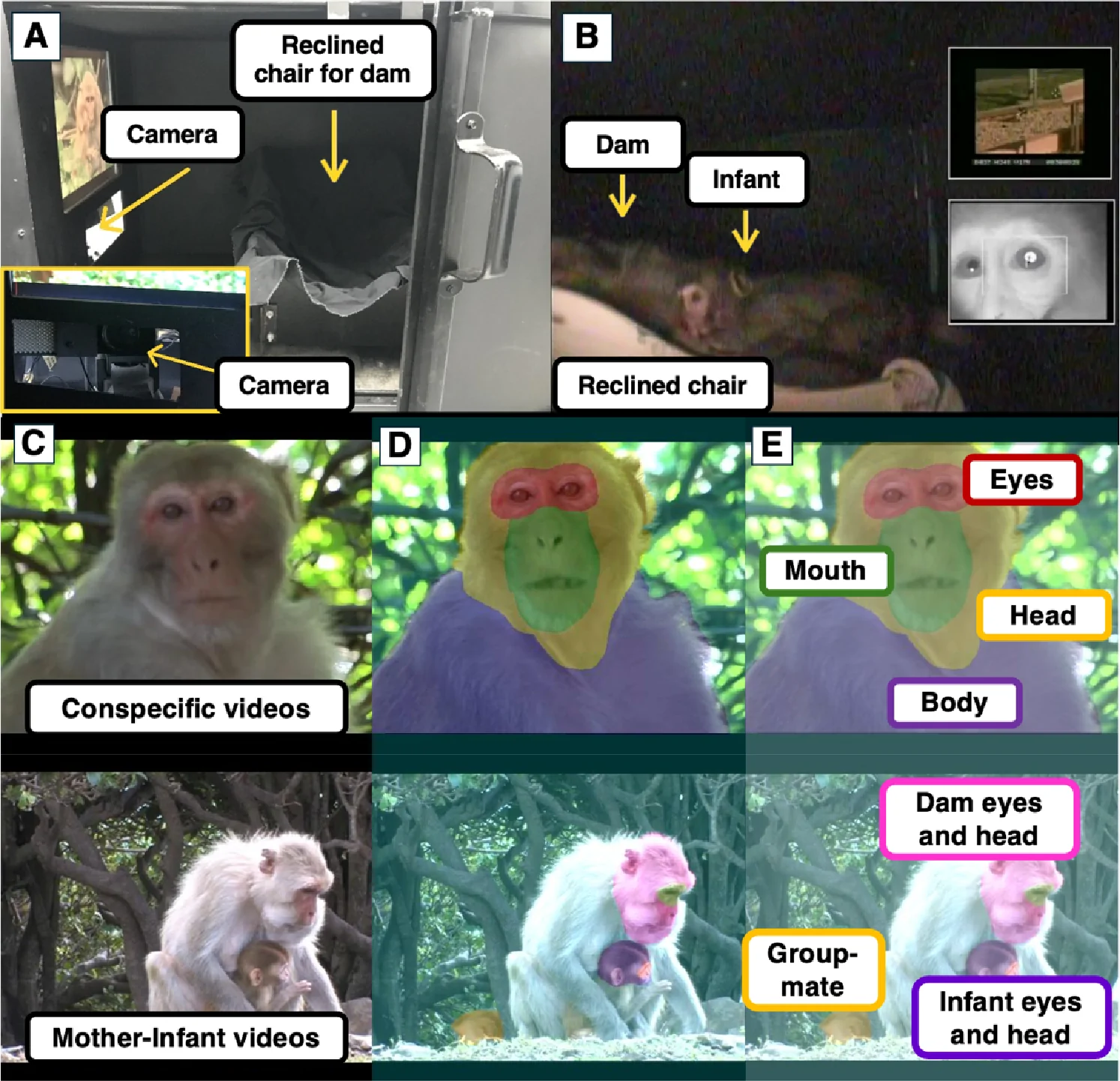
Eye-tracking data collection for infant rhesus macaques and Regions of Interest (ROIs) coding scheme for Conspecific and Mother-Infant video stimuli. (**A**) The testing chamber built for eye-tracking of rhesus macaque infants at the ENBRC, with an adjustable reclining chair for the dam. The infrared camera was mounted on a motorized gimbal and controlled manually by an experimenter during testing. (**B**) Still image of an eye-tracking session in progress. The inset video feed in the upper right corners shows the stimuli being presented and the inset video in the lower right shows the infant eye image. (**C**–**E**) Consecutive frames with ROIs for Conspecific (top row) and Mother-Infant (bottom row) categories of video stimuli (see details in text). Social ROIs in Conspecific and Mother-Infant videos that were used for subsequent analyses are labeled in (**E**).

The sample consisted of 39 typically developing male infant rhesus monkeys (*Macaca mulatta*) that completed eye-tracking while watching social scenes of conspecifics during the first six postnatal months (see *Methods: Subjects & Housing*). Infants were eye-tracked every two weeks from birth to six months (totaling up to 14 sessions) using previously described protocols (see Fig. 1A, B and *Methods: Eye-tracking Procedures* for more details)^6,13–15^. This study only included male infants because it was part of a larger, programmatic initiative to map convergent mechanisms of social learning between humans and non-human primates with relevance for neurodevelopmental conditions associated with social disability, which are more common in males^28^. All procedures were conducted at the Emory National Biomedical Research Center (ENBRC) in Atlanta, United States of America, and were approved by the Institutional Animal Care and Use Committee (IACUC) of Emory University, in accordance with the Animal Welfare Act and the U.S. Department of Health and Human Services “Guide for Care and Use of Laboratory Animals.” The ENBRC is fully accredited by AAALAC, International. Results and Methods are reported in accordance with ARRIVE guidelines^29^.

Two categories of video stimuli were used (Fig. 1): clips showing a single unfamiliar conspecific (39 unique videoclips; referred to hereafter as “Conspecific” videos, see Fig. 1C-E, row 1) and clips of mother-infant interactions (27 unique videoclips; referred to hereafter as “Mother-Infant” videos, see Fig. 1C-E, row 2). Descriptions of video content are included in *Methods: Experimental Stimuli.*

**Fig. 2:**
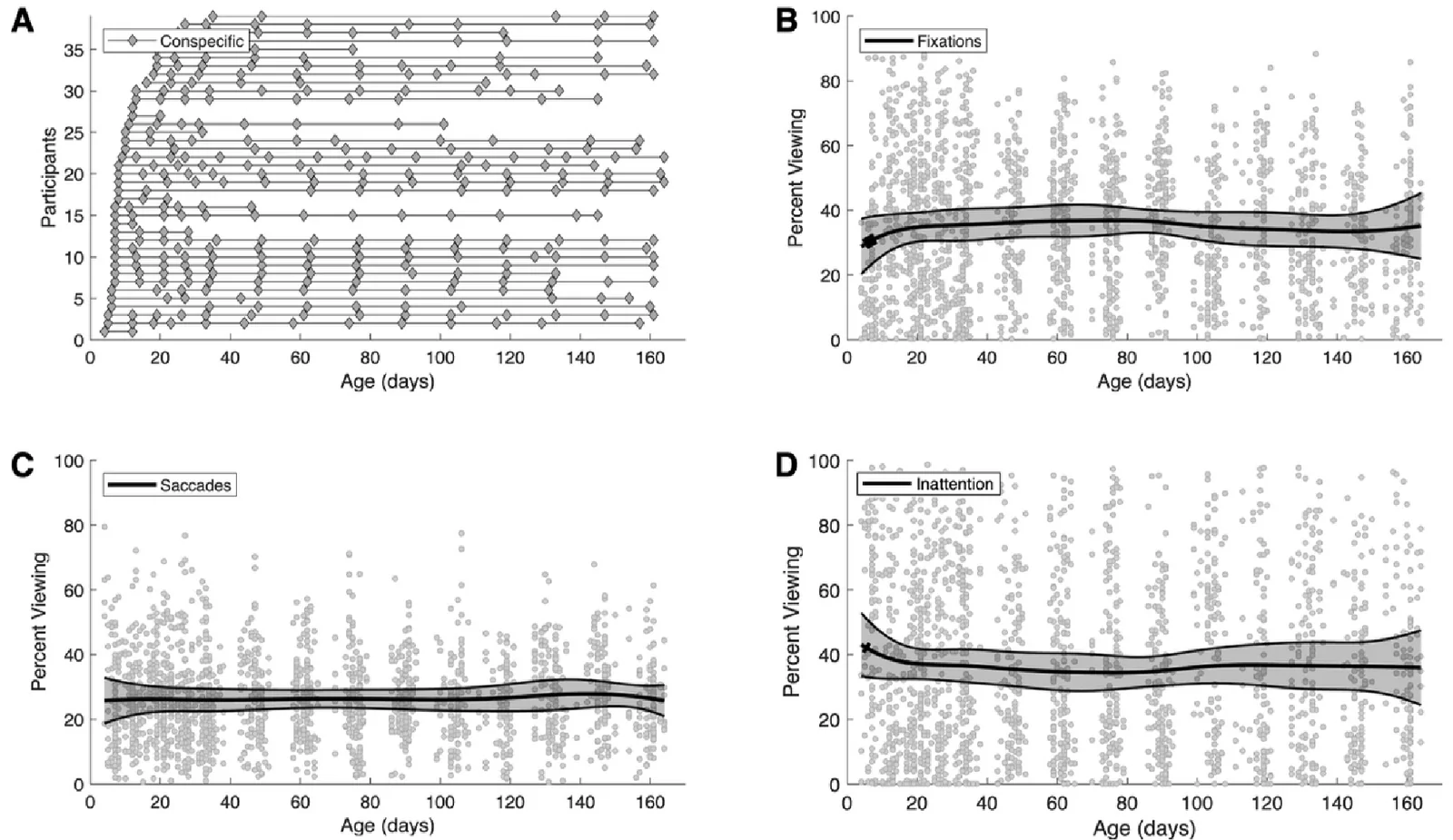
Change in percent of fixations, saccades, and inattention during viewing of Conspecific videos is minimal during the 0–6-month period. Infant macaques (*N* = 39) viewed Conspecific videos at up to 14 timepoints from 0–6 months (4-164 days). (**A**) The sampling distribution of eye-tracking sessions where infants had usable data from Conspecific videos included in analyses. (**B**–**D**) Group mean trajectories of percent fixations, saccades, and inattention during viewing fit using FPCA are banded by 95% confidence intervals and overlaid on raw data. Periods when the rate of change is significantly non-zero (i.e., periods of significant increase or decrease in the group mean) are denoted by thick black lines overlaid on the group mean curve.

**Fig. 3:**
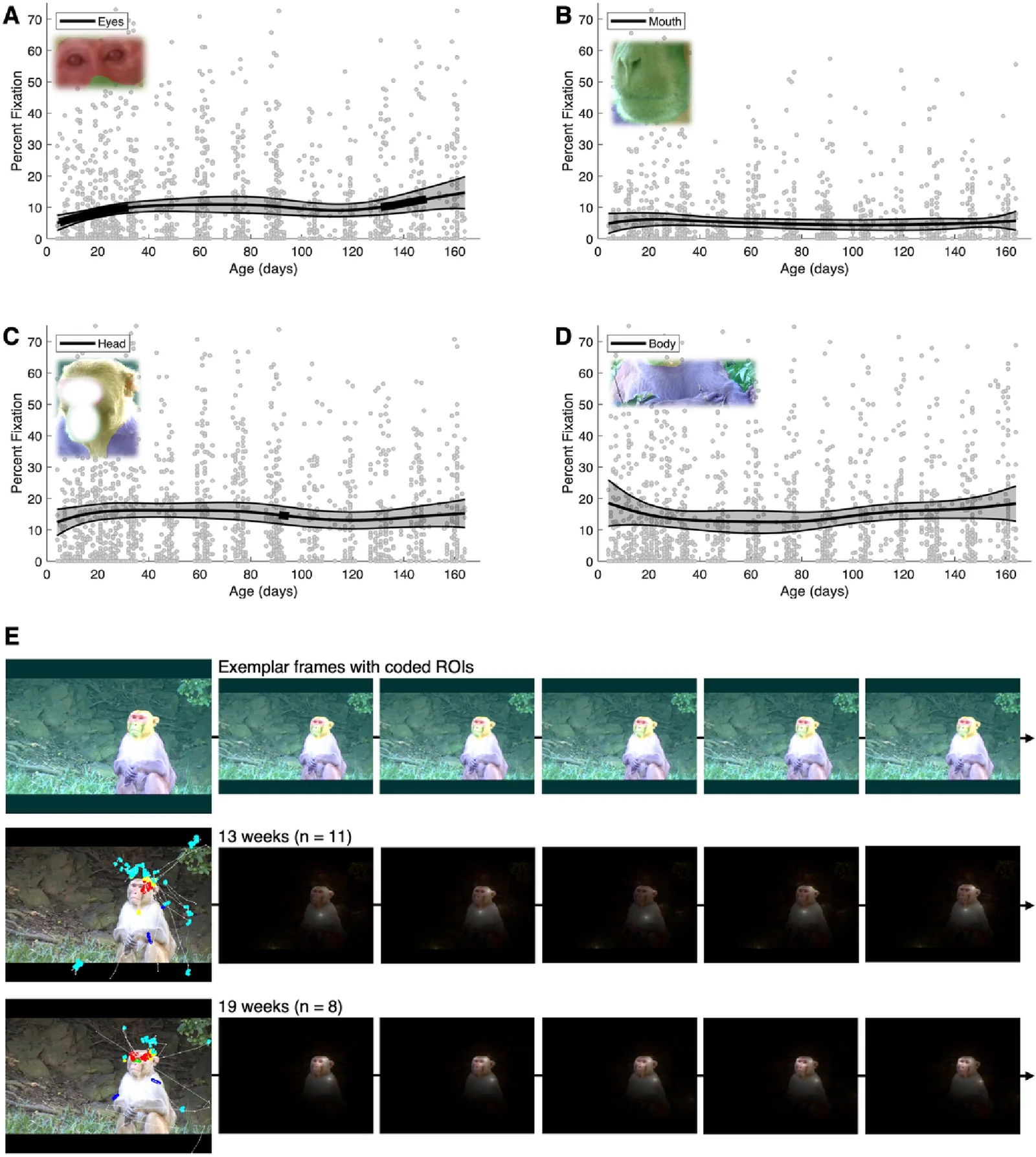
Fixation from 0–6 months to social ROIs defined from the Conspecific videos. A-D, group mean trajectories of attention to the eyes (**A**), mouth (**B**), head (**C**), and body (**D**) of conspecifics during viewing fit using FPCA are banded by 95% confidence intervals and overlaid on raw data. Periods when the rate of change is significantly non-zero (i.e., periods of significant increase or decrease in the group mean) are denoted by thick black lines overlaid on the group mean curve. Images of the ROIs defined from an example frame are included in the plots. The y-axis of all plots has been limited to 75% (range limits of percent fixation are 0-100%) to clearly visualize the trajectories and majority of raw data points. (**E**) Scan paths of individual participants overlaid on example frames highlight group convergence on the Eyes ROI with increasing participant age. Top row: Six example frames from a Conspecific video, color-coded by ROI (see color reference in Fig. 1). Middle and bottom row: First frame image summarizes viewing patterns of N infants who watched the example frames at their 13- and 19-week sessions (solid colors indicate individual fixations color-coded by ROI and thin white lines indicate saccades). Subsequent frame images are overlaid in gray with fixations visualized as transparent points. This approach allows for the visualization of high ‘density’ viewing areas. ROI: Region of Interest.

**Fig. 4:**
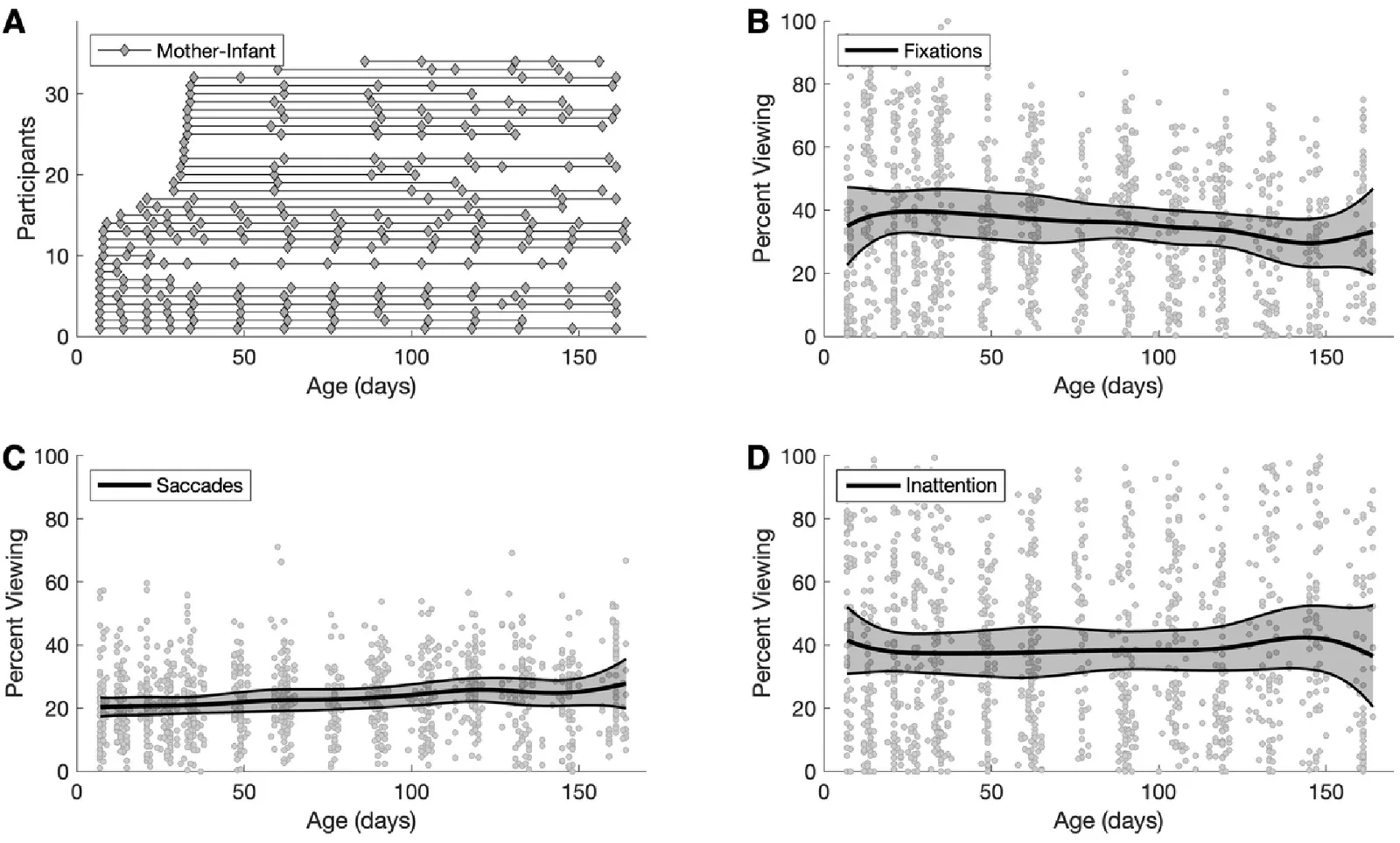
Change in percent of fixations, saccades, and inattention during viewing of Mother-Infant videos is minimal during 0–6-month period. Infant macaques (*N* = 34) viewed Mother-Infant videos at up to 14 timepoints from 0–6 months (7–164 days). (**A**) The sampling distribution of eye-tracking sessions where infants had usable data from Mother-Infant videos. (**B**–**D**) Group mean trajectories of percent fixations, saccades, and inattention during viewing fit using FPCA are banded by 95% confidence intervals and overlaid on raw data. There were no periods of significant increase or decrease in the group means.

**Fig. 5:**
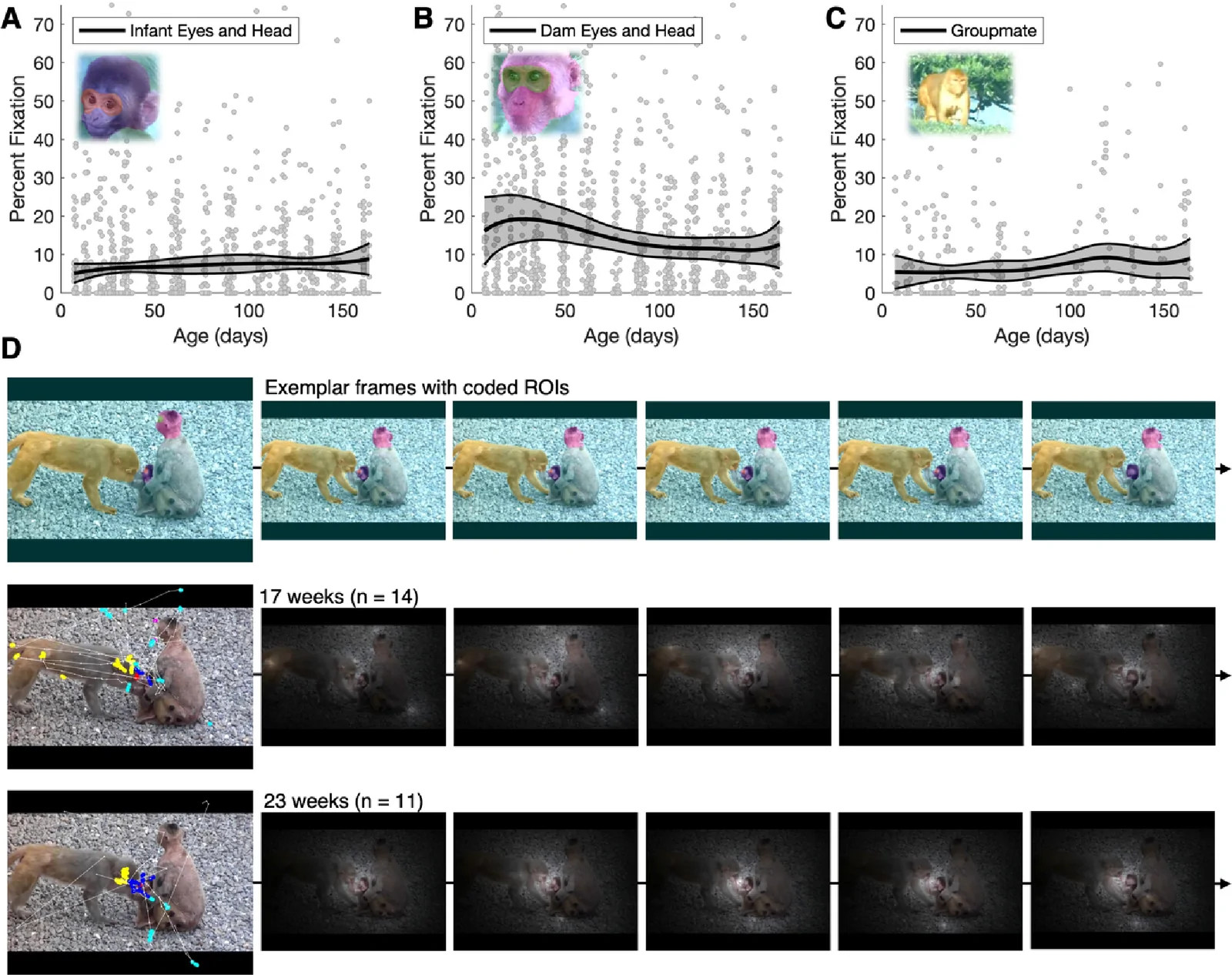
Fixation to ROIs in the Mother-Infant videos from 0–6 months. A-C, group mean trajectories of attention to the Infant eyes and head (**A**), Dam eyes and head (**B**), and background groupmate (**C**) during viewing fit using FPCA are banded by 95% confidence intervals and overlaid on raw data. There were no periods when the rate of change was significantly non-zero (i.e., periods of significant increase or decrease in the group mean). Images of the ROIs defined from an example frame are included in the plots. The y-axis of all plots has been limited to 75% (range limits of percent fixation are 0-100%) to clearly visualize the trajectories and majority of raw data points. (**D**) Scan paths of individual participants overlaid on example frames highlight group convergence on Infant Eyes and Head ROI with increasing participant age. Top row: Six example frames from a Mother-Infant video, color-coded by ROI (see color reference in Fig. 1). Middle and bottom row: First frame image summarizes viewing patterns of N infants who watched the example frames at their 17- and 23-week sessions (solid colors indicate individual fixations color-coded by ROI and thin white lines indicate saccades). Subsequent frame images are overlaid in gray with fixations visualized as transparent points. This approach allows for the visualization of high ‘density’ viewing areas. ROI: Region of Interest.

**Fig. 6:**
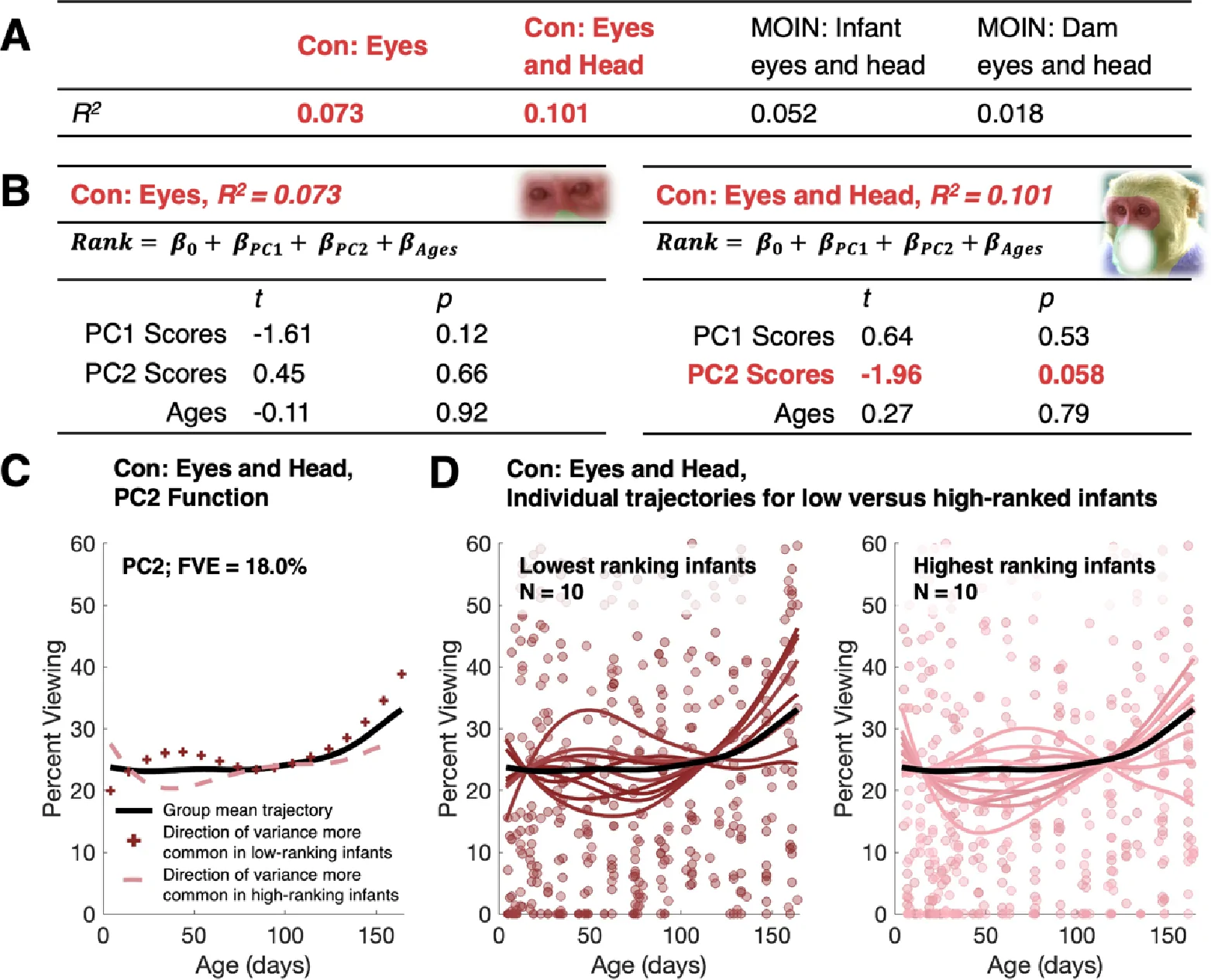
Linear regression models between features from 0–6-months trajectories of attention to eyes and infant social rank. (**A**) regression coefficients for the four linear models tested in this analysis. Column labels (e.g., Con: Eyes) indicate the trajectory of interest. Two models (highlighted in red) showed medium effect sizes (R^2^ > 0.06^32^, and so the beta coefficients for included fixed effects are reported in (**B**). (**B**) Summary statistics from the models highlighted in (**A**). (**C**) The positive and negative directions of variance for the second principal component function (identified in (**B**) as marginally significantly predictive of social rank) visualized around the mean trajectory (solid black line). (**D**) Individual trajectories of attention to the eyes and head of conspecifics for the 10 lowest ranked infants (maroon) and the 10 highest ranked infants (pink) plotted relative to the group mean trajectory (solid black line). FVE: Fraction of Variance Explained.

**Fig. 7:**
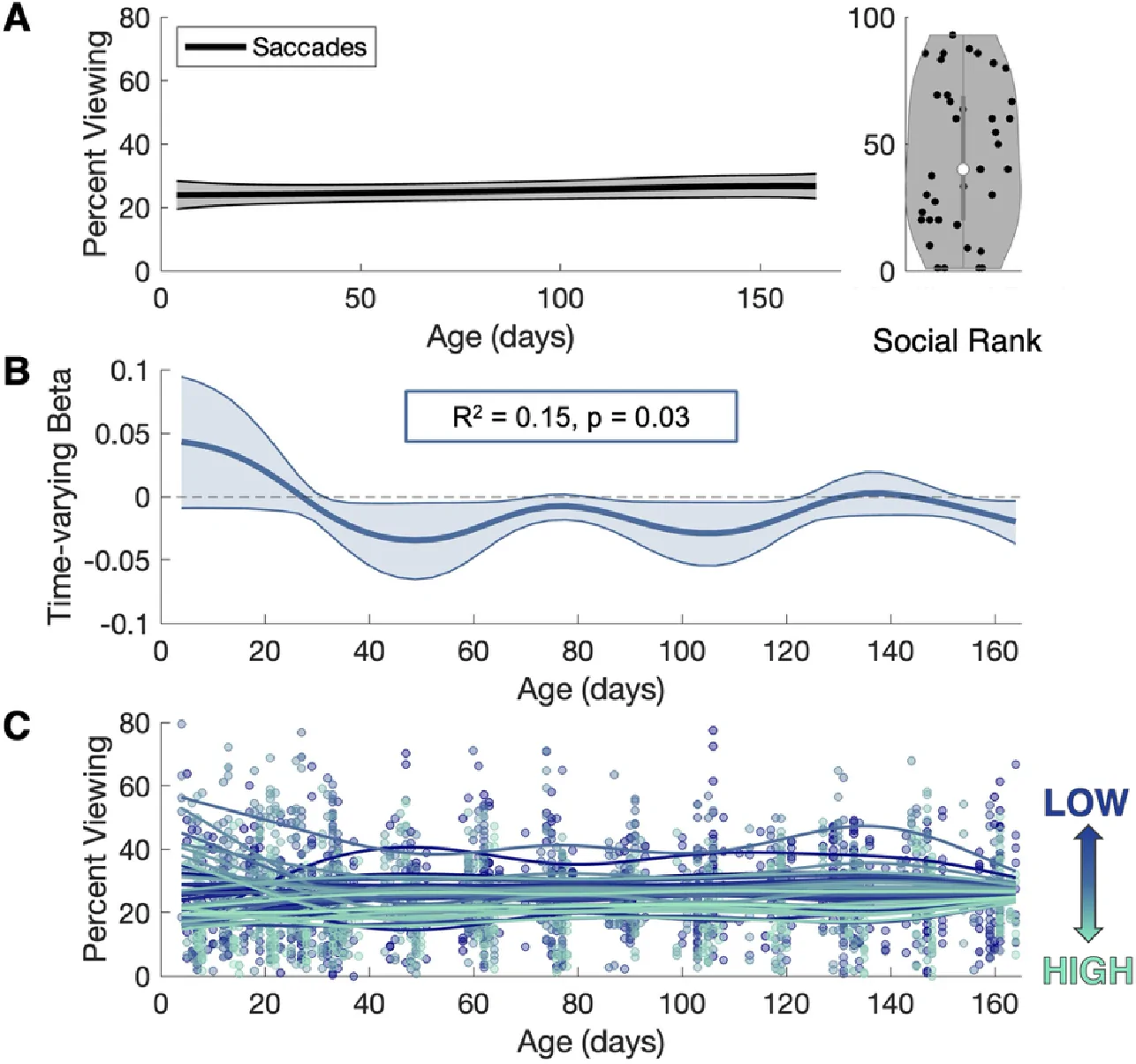
Functional regression model between trajectories of saccades from 0–6 months and infant social rank. (**A**) Plot of the predictor and response variables for the functional regression model. Group mean trajectory of percent of time making saccades fit using FPCA is banded by 95% confidence intervals. Violin plot shows the range and distribution of social ranks among infant participants; gray line shows the interquartile range and white dot shows the median value (**A**). (**B**) Estimated regression parameter surface, beta, for the model banded by 95% pointwise confidence bands. R^2^ and p-value annotated on the plot. (**C**) Individual infant trajectories overlaid on raw data. Trajectories and raw data are color-coded from high to low by infant social rank.

The frequency, defined as percent of total viewing time, with which infant macaques performed oculomotor movements – fixations, saccades, or inattention, defined as data lost due to offscreen looks or movement – was quantified for their viewing of Conspecific and Mother-Infant videos (full list in Table 1), as well as the distribution of infant fixations allocated to the set of social ROIs defined for each video category. For each of these eye-tracking measures, we fitted a group mean curve, generated its first derivative curve, and then derived 95% simultaneous confidence bands for both curves via bootstrap over 1,000 resamples (Figs. 2, 3, 4 and 5). Trajectories of developmental change in the distribution of oculomotor movements and attention patterns to social regions of interest were constructed using Functional Principal Component Analysis – FPCA^30^, see more about FPCA in *Methods: Statistical Analyses*. Associations between features of trajectories of attention to eyes and social rank were tested using linear regression (Fig. 6) and associations between oculomotor measures and social rank were tested using Functional Linear Regression – Scalar (Fig. 7)^31^. All statistical analyses were completed in MATLAB R2024a and R2024b.

**Table 1:** Eye-tracking Measures. Measurements derived from eye-tracking data collection for which change relative to infant age was analyzed using FPCA. ‘N’ refers to the number of infants who contributed data to the Stimulus Type category for analysis and ‘# of Videos’ refers to the number of videos in each Stimulus Type category. Of the 27 Mother-Infant videos with ROIs, 12 videos featured additional conspecifics/groupmates in the scene.

Stimulus type	Eye-tracking measure (%)	*N*	# of videos
*Conspecific*	Fixations to Eyes ROI	39	39
*Conspecific*	Fixations to Mouth ROI	39	39
*Conspecific*	Fixations to Head ROI	39	39
*Conspecific*	Fixations to Body ROI	39	39
*Conspecific*	Fixations Total	39	39
*Conspecific*	Saccades	39	39
*Conspecific*	Inattention	39	39
*Mother-Infant*	Fixations to Dam ROI (Eyes + Head)	34	27
*Mother-Infant*	Fixations to Infant ROI (Eyes + Head)	34	27
*Mother-Infant*	Fixations to Groupmate ROI	34	12
*Mother-Infant*	Fixations Total	34	27
*Mother-Infant*	Saccades	34	27
*Mother-Infant*	Inattention	34	27
*Conspecific and Mother-Infant*	Fixations Total	39	66
*Conspecific and Mother-Infant*	Saccades	39	66

### Analysis of conspecific videos

Seven eye-tracking measures were derived from the Conspecific videos and for each, we constructed developmental trajectories from birth to sixpostnatal months and identified periods when the rate of change in the trajectory significantly differed from zero (as determined by periods when the simultaneous confidence bands for the rate of change curves did not include zero).

#### Trajectories of oculomotor movements during Conspecific videos

The percent of viewing time that infant macaques fixated on or made saccades to content in the Conspecific videos remained relatively unchanged throughout their first six postnatal months (fixations: average of 35.0%, saccades: average of 26.4%) (Fig. 2B-C). The percent of viewing time that they attended off-screen also remained the same (average of 36.2%) (Fig. 2D). The exception to this pattern was in the first postnatal week, when infants showed a slight increase in fixations and decrease in inattention (Fig. 2B, D). The consistency of the distribution of oculomotor movements across early development suggested that these video stimuli held infant attention to the same extent throughout the 0-6-month period.

#### Region-of-interest trajectories from Conspecific videos

Percent fixation to the eyes of conspecifics showed the most dramatic developmental change across the four delineated social ROIs. Just after birth, it started at a minimum of 5.3%, and then significantly increased through the first postnatal weeks to a local maximum of 10.9% at 68 days (approximately 10 weeks), at an average rate of 1.2% a week (Fig. 3A). Percent fixation to the eyes then plateaued, with an observable, though not significant, decrease until 120 days, when it sharply increased (Fig. 3E). This later increase showed a change from 9.1% to 14.6%, which was the maximum value for the 0-6-month period, with a significant rate of increase of an average of 1.6% a week.

The trajectory of percent fixation to the head showed the same general shape as that of percent fixation to the eyes, but with less pronounced changes (Fig. 3C). The maximum percent fixation of 15–16% was maintained from approximately 19–82 days, and then decreased to 13.5% at around 107 days, with a significant rate of -0.7% a week. Percent fixation to the mouth and body of conspecifics showed no periods of significant change from 0-6-months (Fig. 3B, D). Percent fixation to the mouth was maintained at an average of 5.1% and percent fixation to the body, at an average of 14.8%. Though the rate of change was non-significant, percent fixation to the body did show a decrease from 18.0% just after birth to 12.5% at 69 days, which then progressively increased back to 18.2% by the end of the 0-6-month period (Fig. 3D).

### Analysis of mother–infant videos

For each of the six eye-tracking measures derived from the Mother-Infant videos, we constructed developmental trajectories across six months of age and identified periods when the rate of change in the trajectory significantly differed from zero (as determined by periods when the simultaneous confidence bands for the rate of change curves did not include zero).

#### Trajectories of oculomotor movements during Mother-Infant videos

Percent of viewing time fixating on and making saccades to video content, and when the infant was not attending to the video showed no periods of significant increase or decrease, though their trajectories for the Mother-Infant videos were more dynamic than those for the Conspecific videos (Fig. 4B, D). Percent of viewing time spent fixating averaged to 35.4% over the 0-6-month period, with a minimum of 29.6% at 145 days and a maximum of 39.6% at 27 days. Percent of viewing time spent making saccades to onscreen content progressively increased from 20.3% to 27.8% during the 0-6-month period (Fig. 4C). Percent of inattention averaged to 38.8% and dipped to its minimum of 36.5% at day 164 (the last day of measurement). Collectively, these results indicated again that infants are consistently attending to the on-screen content in the Mother-Infant videos in this early developmental period.

#### Region-of-interest trajectories from Mother-Infant videos

Percent fixation to Infant eyes and head and Dam eyes and head (Fig. 5A, B) showed patterns distinct from each other and from percent fixation to eyes from the Conspecific videos (Fig. 3A). Percent fixation to Infant eyes and head showed a progressive increase, though non-significant, of 5.1% to 8.8% from 0 to 6 months (Fig. 5A). This concentrated increase in fixation to Infant eyes and head was showcased by visualizing infant fixations frame-by-frame at 17 weeks (~ 120 days) versus at 23 weeks (~ 160 days): with increasing age there was a clear convergence of infant macaque fixations to the head of the infant on-screen versus to the dam or groupmate (Fig. 5D).

Trajectories of percent fixation to the onscreen Dam eyes and head and the groupmate showed clear patterns of decrease and increase (respectively) over the 0-6-month period, though neither trajectory registered periods with a significantly non-zero rate change. Fixation to Dam eyes and head peaked at 19.2% at 27 days and then progressively decreased to its minimum of 11.1% (Fig. 5B). The confidence bands for fixation to Dam eyes and head were wider than those for fixation to Infant eyes and head, indicating greater individual variability in fixation patterns to that ROI. Fixation to the groupmate began at its minimum of 5.3% and then progressively increased (again, non-significantly) to 8.9% by the end of the study period (Fig. 5C).

An additional analysis was conducted in which attention to Infant eyes and head and Dam eyes and head ROIs was quantified for the 15 videos that did not include the Groupmate ROI to evaluate whether the presence of groupmates in the social scene would influence patterns of social attention (supplemental Fig. 4). The trajectory of attention to Dam eyes and head was highly comparable between videos with and without the Groupmate ROI, but the trajectory of attention to Infant eyes and head was showed context-specific differences. A week-long period of significant increase in attention to the on-screen infant was present from 84 to 93 days and then a longer period of significant decrease from 142 to 162 days (supplemental Fig. 4).

### Analysis of linear regression relationships between trajectories of attention to eyes and infant social rank

#### Infant social rank

The social rank of the infant was defined as the rank of their mother, which is determined by the rank of the matriline within which she is embedded relative to the larger social group. The uniform distribution of normalized social rank ranged from 1% (representing infants in the lowest ranking family of a group) to 92% (representing infants in the highest reported rank of first out of 14 families).

#### Linear regression results

Four models were tested to evaluate whether features of individual trajectories of attention to eyes in the two video types (Conspecific eyes, Infant eyes and head, Dam eyes and head, and Conspecific eyes and head), as indexed through principal component scores (see *Methods: Statistical Analysis*), predicted normalized social rank. Each model included individual scores on the derived principal components (*N* = 2) and the number of ages at which each infant provided useable data as fixed effects (see Fig. 6B for example equations). Of the four models tested, two models had a medium to large effect size^32^: features of trajectories from attention to eyes in the Conspecific videos (Con: Eyes, R^2^ = 0.073) and features of trajectories from attention to eyes and head in the Conspecific videos (Con: Eyes and Head, R^2^ = 0.101) (Fig. 6A). Analysis of the beta coefficients for each model revealed that individual variance relative to the PC2 function from Con: Eyes and Head had a marginally significant (*p* < 0.1) predictive effect on social rank (Fig. 6B). PC2 reflects 18% of individual variance around the mean curve (Fig. 6C, supplemental Fig. 3B) and indicated a variance pattern in which individuals diverge from the mean in two periods (1) 16–74 days (2-10.5 weeks) and (2) after 112 days (16 weeks). During these periods, the trajectories of low-ranking infants tended to be higher than the mean and those of high-ranking infants tended to be lower than the mean (Fig. 6D). The effect was subtle, as visualized in Fig. 6D, but low-ranking infants are shifted towards an earlier peak in attention to eyes and head and more attention overall after 112 days. To note, this principal component function explains only 18% of variance about the mean and so does not manifest as differences in the total amount of eye-looking, rather in a pattern of variance relative to the mean curve over development (Fig. 6D). Within-individual variation remained greater than variation between low versus high-ranking infants.

### Analysis of functional regression relationships between trajectories of frequency of oculomotor movements and social rank

#### Trajectories of frequency of oculomotor movements while watching social scenes

The amount of time that infant macaques spend making different oculomotor movements while watching social scenes provides information about how they navigate visual opportunities for social communication and learning. Since the amount of time that infant macaques spent fixating on content and making saccades to content was highly comparable across development between the Conspecific and Mother-Infant videos (Figs. 2B and C and 4B and C), we opted to combine measures from both video types to test whether infant social rank was related to the frequency of oculomotor movements across the first six months. Percent of viewing time making saccades during Conspecific and Mother-Infant videos exhibited minimal increase or decrease from 0-6-months (average of 25.3%) and no periods registered a non-zero rate of change (Fig. 7A). Percent of time spent fixating on combined video content displayed a significant increase from 5 to 10 days (average of 6.3% per week) and plateaued at approximately 35.2% for the remainder of the 0-6-month period (supplemental Fig. 5).

#### Functional scalar regression results

Combining the data from the Conspecific and Mother-Infant videos doubled the amount of data per infant from birth to six months (an average of 80 observations per infant versus 35 and 48 for the Mother-Infant and Conspecific videos, respectively). This larger dataset permitted us to implement a more complex regression model, Functional Linear Regression – Scalar (FLR-S), to evaluate whether developmental patterns in the frequency of making saccades or fixations over the 0-6-month period predicted individual variation in infant social rank. FLR-S is a multivariate regression tool where the predictor variable is a trajectory fit using FPCA^31^ and the response variable is a scalar measurement (see *Methods: Statistical Analysis – Functional Linear Regression – Scalar Models*). Using FLR-S allowed for the maintenance of non-linearity in the trajectory of interest (i.e., the trajectory of the frequency of saccades or fixations during the videos of social scenes), which is critical when studying dynamic processes in early development and recognizes that the relationship between two variables may change over time, with certain developmental periods marked by a relatively weaker or stronger association. In the two models tested, the trajectory of percent of viewing time making (1) saccades or (2) fixations was the predictor and normalized social rank was the response. The trajectory variable must be included as the predictor to maintain its functional structure and model how the association may change relative to infant age.

The Saccades-Rank model showed a statistically significant and large effect size (defined as 0.14 > R^2^>0.06^32^), with an R^2^ of 0.15 (*p* = 0.033). The Fixations-Rank model showed a modest effect size, R^2^ = 0.07, but was non-significant (*p* = 0.24). The age-varying Beta for the Saccades-Rank model, which reflects age-varying changes in the direction and magnitude of the relationship, captures a transition from 2 to 5 weeks. The Beta became non-zero after approximately 32 days, decreasing to be negative with an average daily value of -0.017 (Fig. 7B). We confirmed this association persists when social rank was assigned as a category (i.e., high, medium, low) versus a scalar value (supplemental Fig. 6). In summary, after five weeks of age, lower ranking infants tend to make more saccades than higher ranking infants while watching other macaques in social scenes (Fig. 7C).

## Discussion

This study leveraged a free-viewing eye-tracking approach in a large and socially-diverse sample of 39 male infant rhesus macaques, in which infants were unrestrained and not sedated, to investigate developmental changes in attention to social content and the extent to which individual variability in patterns of social visual attention was associated with matrilineal social rank. We replicated past findings that infant attention to the eyes of other conspecifics, versus other social content, shows the most developmental change during the first six postnatal months^6,15^. We also found that matrilineal rank was most strongly associated with the amount of time infants spend making saccades, i.e., how infants opt to sample their social visual environments.

The distribution of oculomotor movements (saccades and fixation time) as well as inattention across early development was consistent across the 0–6-month period for both genres of videos, Conspecific and Mother-Infant. These results indicate that all video stimuli similarly engaged infant attention during this developmental period. Infants viewed most videos in the stimuli set more than once across their six months of participation, so this finding, in addition to a clip-specific control analysis (see Methods: *Experimental Stimuli* for details of analysis), suggests that habituation effects minimally influenced our results.

Percent of time spent fixating on the eyes in the Conspecific videos rose to a peak between six and 10 weeks and plateaued between 10 and 17 weeks before rising again through the end of data collection (Fig. 3A). This trajectory shape replicated findings previously published by our group that utilized overlapping, but more socially homogenous samples than the sample included in this study, and comparable longitudinal modeling approaches, i.e., Wang et al.^6^ using LOESS regression and Ford et al.^15^ using FPCA curve-fitting. Percent fixation to the eyes was more variable between individuals from 19 to 23 weeks (as indicated by wider confidence bands around the mean trajectory (Fig. 3A). This period was similarly identified as one of increased variance by Ford et al.^15^, but the shape of the trajectories between that study and this one differed slightly: Ford et al., found a generally flat slope in later infancy, whereas this study and Wang et al.^6^ found a significantly increasing slope. This subtle difference in trajectory shapes may be due to differences in the subject sample between the three studies.

Attention to the eyes of conspecifics significantly increased in the first four postnatal weeks and then again from approximately 18 to 21 weeks (Fig. 3A). Though the same change pattern was demonstrated during both timepoints, the changing social context of the infant could suggest that these increases are adaptively different. In the first postnatal month, infants are engaged in face-to-face interaction with their dam, characterized by infant-directed signaling behaviors like mutual gaze and lip-smacking^33,34^. Thus, neonates may increase their attention to the eyes of conspecifics as part of their emerging experience with reciprocal social communication. In fact, the amount of time neonates spend in face-to-face interaction predicts their sociability with groupmates at two and five months^35^, which raises the intriguing question of whether variability in attention to the eyes specifically during the neonate period may mediate that predictive relationship. The period from two to five months when attention to the eyes plateaus is characterized by a consolidated preference for social information over non-social information^36^ (reviewed in^37^). These changes in social cognition immediately precede the second period of increase in attention to eyes from 18 to 21 weeks. In this period, infants are beginning to wean and establish social relationships in addition to those with their dam^38^. Thus, the second increase in attention to eyes may be related to infants learning to navigate more complex social interactions with novel group members.

The trajectory of percent fixation to the head showed some similarities in shape when compared with percent fixation to the eyes, but with less pronounced changes (Fig. 3C). Fixation to the head rose to a peak in the second month of life and then dropped to a trough at 17 weeks. This pattern is consistent with that for the Eyes ROI, which the Head ROI immediately surrounds. The head may also function separately from the mouth and eye regions as a distinct source of social information, e.g., via ear movements corresponding with facial expressions^39^. Regardless, the relatively sharp increase in attention to the eyes during late infancy appeared to be a developmental feature unique to the eyes. Percent fixation to the mouth and body of conspecifics showed no periods of significant changes from 0-6-months. The developmental changes in the fixation for these two cues in the current study mirrored those reported in the previous two studies. The shape of the trajectory for attention to the head has not been previously reported by our group.

In the Mother-Infant videos, results from which have not been previously reported, fixation to Infant eyes and head rose slightly, but continuously, from one to 23 weeks. This increase was showcased by visualizing infant fixations frame-by-frame at 17 weeks (~ 120 days) versus 23 weeks (~ 160 days), demonstrating a clear convergence of infant macaque fixations to the head of the on-screen infant (Fig. 5D). In contrast, the curve for fixation to Dam eyes and head exhibited an early, slight rise from one to four weeks followed by a decrease that plateaued after 17 weeks (Fig. 5B). This pattern was also observed in the subset of videos where there was not a Groupmate ROI (supplemental Fig. 4C). Percent fixation to Groupmates rose slightly, but steadily, across the entire 0–6-month period, which aligns with a major behavioral transition in the latter half of the first year in which infants become increasingly independent from their dam and integrate with the larger social group^40^.

Quantifying how infants attend to other dam-infant dyads may provide insight into how infants learn about socio-communicative behaviors embedded within dyadic interaction. The first weeks of infant postnatal experience are defined by close contact with their dam, and enriched exposure to affiliative signaling behaviors like mutual gaze and lip-smacking^33^, which has been documented to be used by dams in a ‘mirroring’ context to reinforce infant mouth opening^11^. The rates of these behaviors decrease dramatically after the first postnatal month^33^. Attention to the head and eyes of dam in the Mother-Infant videos peaks within the first month (Fig. 5B), which contrasts with the way infants engage with the eyes of solitary, neutrally expressive adults (Fig. 3A), which peaks later, at approximately eight weeks. In the first postnatal month, infant visual attention may be drawn by cues or behaviors exhibited by dams with nursing infants, like lip-smacking, that support early functional communication and potentially reinforce infant responses and dyadic behaviors like mutual gaze, which are important for social cognitive development. Event-related analyses that relate specific behaviors in infant-dam interaction to infant gaze patterns will be needed to isolate these dynamics. Such an interactive choreography would confer adaptive advantage because there is a well-established link between rich exposure to face-to-face interactions with their dam and later infant sociality and social processing^35,41,42^. Moreover, older macaques classified as having Low-Social personalities show differences in social visual processing tasks including reduced facial recognition and context-specific gaze aversion during infancy^43^.

While this study’s emphasis on naturalistic social stimuli permits for increased ecological validity in mapping trajectories of developmental change in social attention, the range of video stimuli also introduces variability in stimuli features, which may cause some stimulus-driven effects. The use of FPCA curve-fitting approaches, which derive group and individual level patterns by sharing information across individuals, and the shuffling of videos across sessions, mitigate this limitation because signal at any one age is not exclusively driven by infant exposure to a limited subset of videos.

In analyses of the associations between variance features of trajectories of attention to eyes and infant social rank, only variance in individual trajectories related to the second principal component function for attention to the combined ROIs for the eyes and head of conspecifics showed signal as a predictor of social rank (Fig. 6). Variance in individual trajectories related to principal component functions for attention to only the eyes of conspecifics, to Dam eyes and head combined, and to Infant eyes and head combined, did not predict social rank. Therefore, the marginally significant effect reported in^15^ between variance in individual trajectories related to principal component functions for attention to the eyes was not replicated. Interestingly, however, the variance pattern from attention to conspecific eyes and head found in this analysis did replicate the direction of results shown in^15^. Specifically, low-ranking infants tended to have an earlier peak in their individual trajectories and greater overall amounts of attention to the eyes and head of conspecifics in later infancy. To note, individual trajectories of attention to the eyes and head are heterogenous and highly variable. During the periods of 30–100 days and after 120 days, individual values of percent fixation fall within an approximately 30% range, e.g., ranging within 10–35% at day 50 (Fig. 6D). Our results indicate that although a small proportion of this variance may be related to infant rank, the great majority is related to factors not studied in this investigation, which could include infant state during testing as well as individual genetic variation^44,45^.

Percent of time spent making saccades during viewing time was found to be significantly associated with social rank with an effect size of R^2^ = 0.15. Though modest, the magnitude of this effect is consistent with prior work investigating relationships between rank and social visual behaviors in neonates^26^. The age-varying beta curve showed that the coefficient was significantly negative after about five weeks, indicating that lower-ranked infants spent more time making saccades than higher-ranked infants while watching social scenes. This difference may indicate that infants are implementing adaptive visual strategies shaped by the dominance hierarchy inherent to their surrounding social environment. For example, infants may adjust the durations of their fixations relative to their social rank as they watch social scenes. Additional research is needed to investigate this hypothesis and how it may vary relative to different scene content, e.g., fixations to eyes versus scene background, but this hypothesis does align with published findings that low-ranking male infants have significantly shorter fixation durations within the first postnatal month^26^ and lower-ranked adult macaques navigate social scenes using covert attentional mechanisms and rapid “check-looks”^39^, which ensures that they avoid direct stares, and thus, visual cues that could be considered aggressive overtures. Learning and applying this visual strategy in early infancy, and in a way that is scaled by rank, would suggest a highly adaptive visual behavior fine-tuned to infants’ individual social environment.

The age when the direction of the relationship between infant social rank and amount of time making saccades switches and then stays constant, around 4–5 weeks, aligns with other key inflection points in social attention. During this period, infants also reach their early peak in attention to the eyes of conspecifics (Fig. 3A) and increase the amount of time they engage in mutual gaze with conspecifics, particularly their dam^34^. Infant macaques also begin to shorten their fixations towards conspecific faces showing direct gaze beginning at eight weeks^13^ and from 12 to 16 weeks, the velocity of their saccades performed before and after mutual gaze significantly increases^46^. Much of this prior literature has only included male infants, as does this study. Sex-dependent differences in social visual behaviors do appear to emerge after infancy and adolescence, though some context-specific differences are present in infancy. In the first postnatal month, male and female infants spend the same amount of time attending to the faces of other macaques, but when the face is specifically expressive (affiliative or threatening), male infants attend more than female infants^26^. In addition, social rank is associated with fixation patterns in the first month for male infants, but not female infants^26^. Thus, were we to replicate this study with a cohort of female macaques, we might expect similar trajectories of visual behaviors during the first six postnatal months, but diminished effects of social rank on the visual strategies infants are using to navigate social scenes.

This environmentally-attuned social learning occurs amidst rapid, experience-dependent^47,48^ maturation of functional connectivity between primary visual areas^15,49^, as well as other region pairs in the ventral object and dorsal attention pathways^49^. The sensitivity of maturing primary sensory cortices to material and social stressors, like those experienced by lower-ranking macaques, is well-characterized^50,51^, with studies demonstrating general acceleration of neurodevelopmental processes including synaptic proliferation and pruning^51^. In visual cortex, critical periods open and close earlier in rodents exposed to early stress, and their visual acuity on low-frequency visual tasks also matures earlier^52^. In addition to chronic stress exposure, acute, contextual social stress also alters the execution of saccadic eye movements. Saccade velocity is increased when adult humans view emotional faces^53^, and faster processing of low-frequency visual information has been shown to underly that adjustment when individuals view threatening faces^54^. Thus, a model emerges wherein chronic exposure to social stressors due to low social rank may influence the way infant macaques learn to navigate their social environments at the level of both exhibited visual behavior and subsequent neural processing.

Past research has revealed remarkable convergence in social visual attention patterns at the group level between human infants and infant macaques. These findings, confirmed again through the results from this study, have critical relevance for our understanding of how human infants learn from social partners in the first postnatal months, i.e., the period of greatest experience-dependent brain maturation and the age range in which divergence in social behaviors related to neurodevelopmental conditions first emerges^16,55^. The next step in these translational efforts is to extendto the individual level and (i) identify the factors that shape individual variability in how infants curate their visual learning environments and (ii) the mechanisms of action by which those factors drive individual variability. This study highlights social status as one of these factors and motivates further investigation of how elements of that differential experience, e.g., increased stress, maternal protectiveness, access to resources, may affect phylogenetically conserved processes of social attention across primate species.

## Methods

### Subjects & housing

Subjects included *N* = 39 male infants born to dams from the lowest-to-highest matrilineal ranks and assigned to the study over the course of six birthing seasons from 2016 to 2021. All subjects were born and housed at the Emory National Biomedical Research Center (ENBRC) Field Station in Lawrenceville, Georgia, United States of America (RRID: SCR_001914) and reared by their mothers in large, multigenerational social groups (average of 128 individuals, standard deviation of 46 individuals). This provided infants with species-typical social experiences and opportunities to interact with both male and female individuals of varying ages and social status, while controlling for variables like diet and housing. Each social group consisted of multiple families, or matrilines, with a vertical social hierarchy and was housed in outdoor compounds with access to indoor, temperature-controlled quarters. The average dimensions of the indoor and outdoor rooms were: indoor 380 ft^2^, with a range of 370 to 400 ft^2^, and outdoor 10,708 ft^2^, with a range of 6500 to 15,625 ft^2^. All procedures were approved by the Institutional Animal Care and Use Committee (IACUC) of Emory University, in accordance with the Animal Welfare Act and the U.S. Department of Health and Human Services “Guide for Care and Use of Laboratory Animals.” The ENBRC is fully accredited by AAALAC, International.

Inclusion criteria required that all subjects were born to multiparous adult females observed to be competent, non-abusive, and non-neglectful in their care of prior offspring. If the quality of expected maternal care differed after the infant was born, the infant was excluded from the study. All dams were multiparous, but the number of previous births among dams was not standardized. Dams were selected to have as little genetic interrelatedness as possible with other mothers in the study, and there was never more than one infant born to any single dam assigned to the study. Given that social groups at the Field Station typically only have one or two breeding males, it was not possible to assign infants that all had different sires to the study. Dam-infant pairs were also selected from groups known to be relatively socially stable. Subjects were all born full-term and without need of veterinary assistance, weighing a minimum of 450 g at birth. The study included only male infants to allow for greater statistical power given our small sample size, as well as to align with the broader translational goals of the data for human neurodevelopmental research. All infants in the sample were handled and behaviorally tested as previously reported^6,15^. The sample of 39 infants represents a group of infants that are uniformly distributed across the lowest-to-highest matrilineal social ranks and viewed the same sets of video stimuli. Complete subject data on the infants are provided in supplemental Table 1.

Ranking information was provided by ENPRC Colony Management staff based on observations of dyadic agonistic interactions^18,56,57^, between all females in each social group that were greater than three years old. From each observation, the animal that exhibited submissive behavior was ranked lower (subordinate) than the other animal (dominant), e.g., Monkey 1 was considered ranking below Monkey 2 if she invariably responded submissively (withdrawing, grimacing, and fleeing) when Monkey 2 approached and/or acted aggressively towards her. Observations occurred daily and were opportunistic, meaning focal observations on specific animals were not conducted. In addition to scheduled maintenance observations, observations were conducted when specific social group or experimental conditions occurred, including instances of intra-group conflict and reintroduction to the group after removal for experimental assessment. Individual records of submissive behaviors were then collated in a dominance matrix by family and each family ranked relative to the other families (e.g. 1 = alpha, 2 = beta, etc.), following the general organizing principle that members of the highest-ranked family would not exhibit submissive behaviors to members of other families (i.e., the number of submissive behaviors to other members would equal zero) and members of the lowest-ranked family would exhibit submissive behaviors to members of all other families (i.e., the number of submissive behaviors to all other members would be greater than zero). Information about relative family social status within the larger group was collected over a quarterly basis. When significant events occurred that affected the dynamics of the larger group, e.g., a family removal or overthrow of the dominant family, the matrix would be updated immediately. In sum, during the six-month period of infant participation, family status was recorded at birth and then systematically assessed two to three times throughout the study.

Each infant was assigned its mother’s matrilineal social rank, which reflects the status of their family/matriline relative to all families in the social group. A rank of 2/8 would indicate the infant was born into the second-ranking family out of eight total families in that group and is thus a relatively high-ranking individual. Ranks were normalized to a percentile by dividing the family rank by the total number of families, then subtracting this value from one (e.g., 2/8 = 75%), such that percentiles closer to zero represent lower social status and percentiles closer to one represent higher status (supplemental Table 1). Categorical rank (high, medium, low, used for supplemental Fig. 6) was determined by the family being in the upper, middle, or lower third of the group. In compounds with five families, the first and second families were assigned High, and the fourth and fifth families were assigned Low. During the period of data collection, an overthrow occurred in one compound, resulting in rank shifts of three subjects (#’s 43, 44, 57; see supplemental Table 1 for details). We used subjects’ rank at the time of birth for all analyses.

### Eye-tracking procedures

Male infant rhesus macaques were tracked every two weeks over 6 months (1, 2, 3, 4, 5, 7, 9, 11, 13, 15, 17, 19, 21, and 23 weeks postnatally), using procedures previously described^6,13–15^ and briefly summarized below. These protocols were part of a larger programmatic study of neurobehavioral development in infant rhesus macaques.

On the date of testing, the mother-infant pair was removed from their social compound and brought to an onsite behavioral testing facility. The mother was sedated with intramuscular injections of Telazol (4-5 mg/kg), which could be supplemented if needed with Ketamine Hydrochloride (10 mg/kg), and positioned comfortably in a chair inside the custom-built testing chamber. All sedation protocols followed recommendations by ENBRC veterinarians and standard operating protocols, which are in accordance with guidance from the American Veterinary Medical Association and approved by the Emory University IACUC. The dam’s condition was closely monitored throughout the session for signs of movement or distress. The infant remained unrestrained and in ventral contact with its mother for the entirety of the session (Fig. 1A, B) to maintain comfort, though the altered state of the dam may still have impacted infant stress levels. Stimuli were presented on a computer monitor affixed to one wall of the chamber. An infrared camera was mounted immediately below the video monitor on a motorized gimbal, allowing an experimenter to manually track the infant’s eye and adjust zoom and focus throughout the session (Fig. 1A). The infant’s gaze was calibrated at the start of each session using a 5-point procedure (detailed in^16^), then the presentation of the stimuli “playlist” was initiated. Data were collected using technology developed by ISCAN (http://www.iscaninc.com). Any experimental stimuli not attended by the infant were repeated at the end of the session when time permitted. A single session lasted 10–15 min if calibration proceeded quickly and no stimuli were replayed, extending if necessary to a maximum duration of 30 min to limit the mother’s time under sedation. After testing, the mother-infant pair was placed in holding cage in an adjacent room and monitored to allow for full recovery of the mother from sedation. When the mother was fully alert, the pair was returned to their social compound at the Field Station.

### Experimental stimuli

To prioritize ecological validity, high quality videos of freely behaving rhesus macaques located at the ENBRC or the Caribbean Primate Research Center on Cayo Santiago, Puerto Rico were edited to create short clips of approximately ten seconds in length. None of the individual macaques featured in the videoclips were familiar to our subjects. Two categories of video stimuli were used (Fig. 1C-E): Conspecific and Mother-Infant videos.

Each of the Conspecific videos (total *N* = 39 videos) focused on a single monkey exhibiting neutral facial expressions and not vocalizing. Most of the movies contained no or extremely quick instances of direct gaze (typically as the monkey shifted its gaze), and in the few with longer stretches, averted gaze still made up the majority of the gaze pattern. Individual monkeys included a relatively equal distribution of juvenile and adult monkeys, as well as males and females. In videos featuring the adult monkeys, the torso, arms, and head covered approximately 50% of the screen from frame to frame, which dropped to about 40% coverage in videos featuring juveniles given their smaller size. Animals were typically sitting on the ground or in greenery, often eating (9) or observing the environment around them (28).

The Mother-Infant videos (total *N* = 27 videos) contained a relatively limited range of dyadic behaviors on the part of both infant and dam. An additional ROI was parsed for background groupmates in 12 of the videos. These videos were balanced across ages in the playlists, and on average, made up 43.2% (standard deviation of 7.0%) of videos included for analysis at each session. In most videos infant was in close physical contact with the dam, usually nursing (13 videos), nursing asleep (2), or grabbing and/or climbing on the dam while she groomed (7). In five of the videos, infant was not in close physical contact with the dam, but the dam was very close by or restraining infant by tail or foot while it explored the nearby environment. In most videos, the dam was relaxed and observant, either sitting (7), eating (5), or grooming infant (10), typically its face or hands. Six videos contained examples of mutual gaze, with the majority between dam and infant (4) and in two instances, between infant and a nearby juvenile while infant maintained contact with dam. The presence of a groupmate or groupmates in the background did not change the distribution of dyadic behaviors exhibited in that subsample of 12 videos. The number of groupmates in the background ranged from one to three and in most videos (9), they did not interact with the dam-infant pair. In these videos, groupmates were typically other adults, and in two videos, included another dam-infant pair. In three of the videos, there was an additional juvenile macaque that did interact with the infant (2) or dam (1).

Within a given session, approximately 30% of the videos shown were novel and 70% had been shown in at least one previous session. Each unique Conspecific and Mother-Infant videoclip was included in an average of three and no more than six sessions. To assess whether infants may have habituated to video stimuli shown at consecutive visits, we performed a clip-specific control analysis. The highest density of visits occurred in weeks 1–6, when infants had weekly sessions, so habituation effects, if any, were expected be strongest in that period. Eight infants watched the same clip at visits 1, 2, and 3. To assess if they habituated to the content, indexed by a greater amount of inattention to that clip, a linear mixed model was used to test if amount of inattention was predicted by session number, with infant ID added as a random effect. The model was appropriately fit to the gaussian distribution (evaluated through a Kolmogorov-Smirnov test, D = 0.01, *p* = 0.97), and the standardized coefficient for session number was not significantly different from 0 (β =-0.081, t=-1.33, *p* = 0.20). This result shows that the amount of inattention did not increase or decrease for this exemplar clip though infants saw it three weeks in a row. Importantly, this clip was the only one that infants saw in 2 + consecutive sessions a week apart – all other clips were seen only at two weekly sessions or between sessions two weeks apart.

During testing, the two categories of videos were interspersed with other stimuli categories that are not discussed in this paper. Additional content included (i) dynamic videos of additional social scenes, specifically social group grooming and/or play, and (ii) videos of static photos that represented comparative paradigms like direct versus inverted gaze, upright versus inverted head position, and scrambled versus unscrambled faces. The order of videoclip presentations was pseudorandomized with all subjects viewed the same playlists at the same ages, but in differing orders of presentation.

### ROI analysis

For the Conspecific videos, regions of interest (ROIs) were previously created by Wang et al.^6^ to capture: total screen area, body, head, eyes, and mouth (Fig. 1C-E, row 1). To analyze the Mother-Infant videos, we created ROIs to capture six categories of content: total screen area, infant’s eyes, dam’s eyes, infant’s head, and dam’s head, with an additional ROI for other rhesus macaques appearing in the scene, referred to hereafter as “groupmates” (Fig. 1C-E, row 2). ROIs were hand traced frame by frame, using in-house software written in MATLAB (v. R2018b), and stored as binary bitmaps. For subsequent analyses, the ROIs for Dam eyes and head, and for Infant eyes and head, were combined to create two ROIs for “Dam eyes and head” and “Infant eyes and head,” due to the small size of the eye region ROIs (Fig. 1E) and low percent fixation.

In-house software in MATLAB (v. R2018b) was used to automatically parse the data recorded during eye-tracking sessions into four distinct types of eye movements: fixations, saccades, blinks, and inattention, i.e., data lost due to offscreen looks and movement. A fixation was counted whenever the infant’s gaze rested in the same location on the screen for two frames consecutively (or one-fifteenth of a second given the frame rate of 30 Hz). Each fixation was further tagged with a specific ROI. Saccades were defined by the velocity of eye movements, based on a threshold of 30 degrees/sec. Saccades and blinks were not tagged to ROIs. Percentages of fixations, saccades, and inattention relative to the number of frames (typically around 300 frames) were calculated for each video as well as percentages of fixation to each predetermined ROI, relative to the total count of fixations for the entire videoclip.

Data were excluded if zero fixations were counted during the presentation of the videoclip. We note that this criterion differed from the exclusion threshold of 20% fixations (approx. 60 frames) applied in some previous eye-tracking studies (e.g.^6,15,16^), . We chose a less stringent criterion for the current analyses because we were interested in specific ROIs and in global measures of viewing behavior and data quality. We also chose this criterion to avoid the risk of excluding potentially meaningful datapoints, given that monkeys are known to show overall poorer attention towards video stimuli, compared to human participants, and to perform more saccades and covert viewing strategies like brief “check-looks” to the screen, rather than overt fixation^6,39,58^.

### Statistical analysis

#### Functional Principal Component Analysis (FPCA)

Flexible mapping of change processes is key to testing and establishing accurate models of infant development^59,60^. Functional data analysis tools were used to describe and quantify features of developmental change itself because trajectories can be influenced by factors entirely separate from those that influence outcome measures at any one timepoint^61^. To identify key derivative features of developmental trajectories in social-visual attention, such as rates of change and the timing of inflection points, we generated longitudinal growth curves using Functional Principal Component Analysis (FPCA). FPCA is a non-parametric, data-driven approach to curve-fitting that does not require making *a priori* assumptions about a curve’s true underlying shape.

Group mean and individual trajectories were generated for each measure of social visual attention derived from the video content (Table 1). Parameter selection for curve-fitting was done separately for each measure and smoothing parameters were selected to optimize the final curve shape derived from applying a Gaussian filter to the data (see supplemental Table 2). Variance around the group mean trajectory was estimated by computing 95% simultaneous confidence bands. Rates of change for each measure were calculated by taking the first derivative of the modeled mean curve and periods of significant rates of change were identified as windows when the 95% simultaneous confidence bands around the rate of change curve did not include 0.

Orthogonal directions of variance in the raw data relative to the mean curve were quantified as principal component functions that explained some percent of variance about the mean curve over infant age (up to 100%) and, for each function, a ‘score’ was generated for each participant that quantified the extent to which their individual trajectory followed the shape of that direction of variance. Individual scores for principal component functions were used in linear regression models to evaluate how features of individual trajectories of attention to the eyes in either Conspecific or Mother-Infant videos predicted social rank.

#### Linear Regression Models

Linear regression models were constructed to evaluate how features of individual trajectories (quantified as scores on principal components) predicted infant social rank for four measures of attention to eyes: percent fixation to conspecific eyes in the Conspecific videos, to Dam eyes and head in the MOIN videos, to Infant eyes and head in the MOIN videos, and to allow for more direct comparisons between video types, to conspecific eyes and head in the Conspecific videos. Four total models were tested, and results are presented in *Results: Analysis of linear regression relationships between trajectories of attention to eyes and infant social rank* and Fig. 6. The group mean trajectory for each measure had up to two principal components so individual scores for each component were included as fixed effects in each regression model. The robustness of individual trajectories, which affects the calculation of component scores, is related to the number of longitudinal data points for each individual, and so number of unique ages with usable data was also included as a fixed effect in the models. All models used a Gaussian data distribution and the normality of residuals was confirmed using Kolmogorov-Smirnov tests: (i) Con: Eyes model, D = 0.10, *p* = 0.75, (ii) Con: Eyes and Head, D = 0.10, *p* = 0.79, (iii) MOIN: Infant Eyes and Head, D = 0.12, *p* = 0.72, and (iv) MOIN: Dam eyes and head, D = 0.099, *p* = 0.86. Additional confirmation of model fit for models reported in Fig. 6 was determined from plots of fitted values against residuals of predicted values (supplemental Fig. 1). Beta coefficients from models with an R^2^ over 0.06, signaling a medium effect size^32^, are reported.

#### Functional Linear Regression – Scalar Models

In FLR-S, the predictive strength of the model is conveyed via a functional R^2^^31,62^ that specifies how much variance in the response is explained by the entire trajectory of the predictor, and the significance of the model is evaluated via non-parametric bootstrap over 5,000 samples. In functional regression models, the direction and magnitude of the regression estimate, Beta, varies along the timeline of the predictor trajectory, allowing for inference of how the predictive relationship changes with time. Confidence bands around the Beta curve are generated using non-parametric bootstrap over 5,000 samples and can be used to identify periods when Beta is non-zero.

In this study, trajectories of social attention measures are modeled as the predictor and matrilineal social rank is modeled as the scalar response. We combined fixation and saccade information across video types (Table 1) to construct trajectories of percent of viewing time fixating to content and making saccades while watching both Conspecific and Mother-Infant videos. Confirmation of model fit for the model reported in Fig. 7 was determined by plotting fitted values against residuals of the predicted values (supplemental Fig. 2A) and from standard regression diagnostics extended to the functional case (supplemental Fig. 2B, C)^63^.

## Supplementary Information

Below is the link to the electronic supplementary material.

Supplementary Material 1

## Data Availability

Deidentified subject data is available at [https://osf.io/pn647/overview? view_only=c75c07fd5c7042b7b0ee67e54e804a30](https:/osf.io/pn647/overview? view_only=c75c07fd5c7042b7b0ee67e54e804a30) . Data includes demographic information per subject (normalized and categorical social rank) and summary data for eye-tracking measures per subject per session (means and standard deviations).
