# Supplementary material for: Matrilineal social rank is associated with patterns of attention to social scenes in male infant rhesus macaques
Source: Sci Rep. 2026 Oct 21;16:33200. doi: 10.1038/s41598-026-61421-w (PMC13686938; doi:10.1038/s41598-026-61421-w)
Supplement: Supplementary file 1 [file 41598_2026_61421_MOESM1_ESM.docx]

**Supplemental information for:**

**Matrilineal social rank is associated with patterns of attention to social scenes in male infant rhesus macaques**

A. Ford^1,2,*^, N.R. Pilgeram^3,4^, A. Wang^4^, M. C. Alvarado^3^, T. Jonesteller^3^, J. Wesson^3^, K. Bailey^3^, S. Ginsberg^5^, P. Karur^5^, M. Sanchez^3,6^, J. Bachevalier^3,4^

1-Department of Pediatrics, Emory University, School of Medicine, Atlanta, GA, USA

2-Marcus Autism Center; Children’s Healthcare of Atlanta, GA, USA

3-Emory National Primate Research Center, Emory University, Atlanta, GA, USA

4-Department of Psychology, Emory University, Atlanta, GA, USA

5-Department of Neuroscience and Behavioral Biology, Emory University, Atlanta, GA, USA

6- Department of Psychiatry & Behavioral Sciences, Emory University, School of Medicine, Atlanta, GA, USA

Scientific Reports

Contents:

***Supplemental materials – Subject information***

*a) Detailed information about study subjects*

- *Table 1*

***Supplemental methods – Curve-fitting models from FPCA***

*a) Parameter selection for FPCA*

- *Table 2*

***Supplemental methods – Regression model selection***

*a) Model diagnostics for linear regression models (Main Text Fig. 6)*

- *Figure 1*

*b) Model diagnostics for functional scalar regression model (Main Text Fig. 7)*

- *Figure 2*

***Supplemental results***

*a) Principal component functions for trajectories of attention to stimulus categories containing eyes*

- *Figure 3*

*b) Trajectories of percent time fixating to social ROIs in N=15 Mother-Infant videos without Groupmate ROIs*

- *Figure 4*

*c) Trajectory of percent of time spent fixating on content during Conspecific and Mother-Infant videos*

- *Figure 5*

*d) Percent of time spent making saccades predicts matrilineal rank when rank is broadly categorized as High, Medium, or Low versus normalized as a percentile.*

- *Figure 6*

**Supplemental materials – Subject information**

*a) Detailed information about infant subjects*

| **Animal Number** | **Cohort Year** | **Social Group** | **Social Rank at Birth** | **Social Rank (Percentile)** |
| --- | --- | --- | --- | --- |
| 39 | 2021 | #2 | 2 of 14 | 85.7% |
| 40 | 2021 | #5 | 1 of 3 | 66.7% |
| *******45 | 2021 | #1 | 1 of 7 | 85.7% |
| 47 | 2021 | #3 | 4 of 11 | 63.6% |
| *******48 | 2021 | #2 | 1 of 14 | 92.9% |
| 53 | 2021 | #3 | 2 of 11 | 81.8% |
| 52 | 2020 | #1 | 1 of 7 | 85.7% |
| 55 | 2020 | #3 | 8 of 11 | 27.3% |
| 41 | 2019 | #3 | 5 of 11 | 54.5% |
| 42 | 2019 | #3 | 9 of 11 | 18.2% |
| 43 | 2019 | #6 | 5 of 5 | 1.0% |
| 44 | 2019 | #6 | 4 of 5 | 20.0% |
| 57 | 2019 | #6 | 4 of 5 | 20.0% |
| 46 | 2018 | #3 | 11 of 11 | 1.0% |
| 49 | 2018 | #1 | 1 of 8 | 87.5% |
| *******50 | 2018 | #4 | 5 of 8 | 37.5% |
| 51 | 2018 | #1 | 8 of 8 | 1.0% |
| 56 | 2018 | #6 | 1 of 6 | 83.3% |
| 22 | 2016 | #8 | 2 of 5 | 60.0% |
| 23 | 2016 | #6 | 2 of 5 | 60.0% |
| 9 | 2016 | #9 | 7 of 10 | 30.0% |
| 10 | 2016 | #6 | 3 of 5 | 40.0% |
| *******25 | 2016 | #7 | 3 of 5 | 40.0% |
| 11 | 2016 | #1 | 10 of 10 | 1.0% |
| 26 | 2016 | #4 | 6 of 10 | 40.0% |
| 12 | 2016 | #8 | 4 of 5 | 20.0% |
| 27 | 2016 | #4 | 3 of 9 | 66.7% |
| 28 | 2016 | #1 | 7 of 10 | 30.0% |
| 29 | 2016 | #2 | 2 of 3 | 33.3% |
| 15 | 2016 | #3 | 12 of 13 | 7.7% |
| 16 | 2016 | #7 | 3 of 5 | 40.0% |
| 17 | 2016 | #1 | 2 of 10 | 80.0% |
| *******58 | 2016 | #3 | 10 of 11 | 9.1% |
| *******31 | 2016 | #3 | 10 of 13 | 23.1% |
| *******32 | 2016 | #1 | 9 of 10 | 10.0% |
| *******35 | 2016 | #1 | 5 of 10 | 50.0% |
| 20 | 2016 | #6 | 2 of 5 | 60.0% |
| 37 | 2016 | #3 | 4 of 13 | 69.2% |
| *******38 | 2016 | #3 | 4 of 13 | 69.2% |

**Table 1| Subjects’ demographics.** Social rank and year of birth for infant subjects (*N* = 39). The numerical social ranks listed here represent the status of the infant’s family/matriline within their social group, rather than the relative rank of the individual dam out of all individuals in the social group. The rank percentile was adjusted to 1% from 0% for Subjects 11, 43, 46, and 51. Nine subjects – six infants from the 2016 cohort, one from the 2018 cohort, and two from the 2021 cohort - were removed from the study before reaching 6 months of age due to health concerns, injury, maternal rejection, inability of equipment to accommodate the dam’s size, or instability in the social group hierarchy. These subjects are indicated by asterisks (*******). The greatest drop-off in subjects (*N* = 9) occurred in the first 1 to 5 weeks of life. An overthrow took place in group #6 when Subjects 43, 44, and 57 were around 8 weeks (56 days) old, the result of which was Subjects 44 and 57’s family dropping from 4th-ranked to 5th-ranked of five families, while Subject 43’s family rose from 5th-ranked to 3rd-ranked. For further information on the 2016 cohort, see Wang et al. (2020) and Ford et al. (2023).

**Supplemental methods – Curve-fitting models from FPCA**

*a) Parameter selection for FPCA*

Smoothing parameters were selected to minimize error between estimated participant curves and their processed data and to reflect developmentally appropriate shapes – i.e., based on prior work (Wang et al. 2020, Ford et al. 2023), we would not expect fixation to eyes to drop steeply at any point along this trajectory. Smoothing parameters were also adjusted to minimize fitting artifacts like boundary effects.

|  | $\boldsymbol{w}_{\boldsymbol{\mu}}$ | $\boldsymbol{w}_{\boldsymbol{G}}$ | **Eigenfunctions** | **FVE** |
| --- | --- | --- | --- | --- |
| ***Conspecific*** |  |  |  |  |
| Saccades | 15 | [34, 34] | 2 | 92.0%, 7.35% |
| Lost/offscreen attention | 15 | [34, 34] | 2 | 82.7%, 13.4% |
| Total fixations | 15 | [36, 36] | 2 | 87.3%, 10.3% |
| Eye fixations | 18 | [28, 28] | 2 | 72.6%, 27.4% |
| Mouth fixations | 15 | [34, 34] | 2 | 87.4%, 12.4% |
| Head fixations | 18 | [30, 30] | 2 | 82.9%, 14.8% |
| Body fixations | 19 | [32, 32] | 2 | 82.5%, 17.4% |
| ***Mother-Infant*** |  |  |  |  |
| Saccades | 13 | [32, 32] | 1 | 96.28% |
| Lost/offscreen attention | 16 | [36, 36] | 2 | 92.4%, 5.9% |
| Total fixations | 13 | [34, 34] | 2 | 52.2%, 43.7% |
| Infant eyes and  head fixations | 20 | [36, 36] | 1 | 95.3% |
| Dam eyes and  head fixations | 14 | [30, 30] | 2 | 85.0%, 14.6% |
| Groupmate  fixations | 20 | [32, 32] | 2 | 92.7%, 4.6% |
| ***All videos*** |  |  |  |  |
| Saccades | 25 | [12, 12] | 2 | 85.6.4%, 10.2% |
| Total fixations | 12 | [18, 18] | 2 | 54.9%, 40.8% |

**Table 2**| **Parameter values for FPCA curve-fitting for eye-tracking measures.** $w_{\mu}$ specifies the smoothing bandwidth for the mean function, $w_{G}$ specifies the smoothing bandwidth for the covariance surface. FVE specifies the percent of variance explained by each eigenfunction, the total number of which are specified in Eigenfunctions. Abbreviations: FVE – Fraction of Variance Explained.

**Supplemental methods – Regression model selection**

*a) Model diagnostics for linear regression models (Main Text Fig. 6)*

The distribution of residuals for the Con: Eyes and Con: Eyes & Head linear models reported in Figure 6 and *Results: Analysis of linear regression relationships between trajectories of attention to eyes and infant social rank* was evaluated by plotting the fitted versus residuals of the predicted values (Fig. 1). Neither model shows major effects of heteroscedasticity nor non-linear relationships between the fitted and residual values, indicating that the assumptions of using a linear model to predict infant social rank from features of trajectories of attention were not violated.

**Figure 1| Fitted values plotted against residuals of predicted values for linear regression models reported in Main Text Fig. 6B.** Fitted versus residual values for the Con: Eyes (**A**) and Con: Eyes & Head models (**B**) (equations in Main Text Fig. 6B).

*b) Model diagnostics for functional scalar regression model (Main Text Fig. 7)*

The diagnostics for the functional model aiming to predict infant social rank from trajectories of percent viewing time spent making saccades (Main Text Fig. 7) were implemented as recommended by Chiou & Müller (2007). This approach included plotting the fitted versus residual values (Fig. 2A) to evaluate if the final model complied with relevant assumptions and evaluating functional leverage and functional Cook’s Distance values by individual to identify potential outliers who may be driving the effect (Fig. 2B,C).

The model did not show major effects of heteroscedascity or non-linear relationships between the fitted and residual values (Fig. 2A). Functional leverage and Cook’s Distance values showed that while no individuals had a value greater than 1, which is used as a general indicator of an overly influential outlier, three individuals (marked a, b, c, in Fig. 2) were identified to exert greater influences than others as indicated using a more stringent test in which individual values are compared against three times the value of the mean across all individuals. To confirm robustness, these three individuals were removed from the sample, and the model was re-run with the original parameters. The effect was confirmed in the smaller sample (R^2^ = 0.29, p = 0.005). Since their influence was only detected using the more stringent approach and their inclusion reduced the potential for heteroscedasticity at the whole sample level, all three individuals were included in the final model presented in this study (Main Text Fig. 7).

**Figure 2| Diagnostics for functional scalar regression model reported in Main Text Fig. 7. A,** Fitted versus residual values. **B**, functional leverages for each individual in the model. **C**, functional Cook’s Distance values for each individual in the model. a, b, c, denote three individuals who were identified as potentially having more influence on model performance.

**Supplemental results**

*a) Principal component functions for trajectories of attention to stimulus categories containing eyes*

**Figure 3| Principal component (PC) functions for trajectories of attention to stimulus categories containing eyes from Conspecific (Con) and Mother-Infant (MOIN) videos. A-D**, plots of the mean curve and PC functions around the mean curve for four categories of social ROIs. The first column shows the mean curve (thin black line) plotted over the raw data. The y-axis of all plots has been reduced (range limits of percent fixation are 0-100%) to clearly visualize the trajectories and majority of raw data points. The second and third column show the positive and negative directions of the first and second, respectively, PC functions plotted around the mean curve. The + signs denote the trajectory shape more common to participants with a positive PC score relative to that component and the – signs, the trajectory shape more common to participants with a negative PC score. **C**, only one PC function explained most of the variance of individual data relatively to the mean trajectory for MOIN: Infant Eyes and Head.

*b) Trajectories of percent time fixating to social ROIs in N=15 Mother-Infant videos without Groupmate ROIs*

**Figure 4| Fixation to Infant and Dam ROIs in the Mother-Infant videos without a Groupmate ROI from 0-6 months.** Infant macaques (N=32) viewed this subset of Mother-Infant videos at up to 14 timepoints from 0-6 months. **A**, the sampling distribution of eye-tracking sessions where infants had usable data from subset of Mother-Infant videos. **B-C**, group mean trajectories of attention to the Infant eyes and head (**B**), Mother eyes and head (**C**), during viewing fit using FPCA are banded by 95% confidence intervals and overlaid on raw data. Periods when the rate of change is significantly non-zero (i.e., periods of significant increase or decrease in the group mean) are denoted by thick black lines overlaid on the group mean curve. Images of the ROIs defined from an example frame are included in the plots. The y-axis of all plots has been limited to 75% (range limits of percent fixation are 0-100%) to clearly visualize the trajectories and majority of raw data points.

*c) Trajectory of percent of time spent fixating on content during Conspecific and Mother-Infant videos*

**Figure 5| Change in percent of fixations during viewing of Conspecific and Mother-Infant video.** Infant macaques (N=39) viewed Conspecific and Mother-Infant videos at up to 14 timepoints from 0-6 months. Group mean trajectory of percent fixations during viewing fit using FPCA is banded by 95% confidence intervals. Periods when the rate of change is significantly non-zero (i.e., periods of significant increase or decrease in the group mean) are denoted by thick black lines on the group mean curve.

*d) Percent of time spent making saccades predicts matrilineal rank when rank is broadly categorized as High, Medium, or Low versus normalized as a percentile.*

**Figure 6| Functional regression model between trajectories of saccades from 0-6 months and categorical social infant rank (*Methods: Subjects & Housing*).** **A**, plot of the predictor and response variables for the functional regression model. Group mean trajectory of percent of time making saccades fit using FPCA is banded by 95% confidence intervals. Violin plot shows the levels of categorical social rank among infant participants. **B,** shows estimated regression parameter surface, beta, for the model banded by 95% pointwise confidence bands. R^2^ and p-value annotated on the plot. **C,** shows individual infant trajectories overlaid on raw data. Trajectories and raw data are color-coded as high, medium, or low by infant matrilineal rank.

References:

Chiou, J.-M., & Müller, H.-G. (2007). Diagnostics for functional regression via residual processes. Computational Statistics & Data Analysis, 51(10), 4849–4863. https://doi.org/10.1016/j.csda.2006.07.042
